# Delayed Chyle Leak Following Anterior Cervical Spinal Surgery: A Case Report and Management Algorithm

**DOI:** 10.7759/cureus.1231

**Published:** 2017-05-09

**Authors:** Kyle Mueller, Hasan R Syed, Jay W Rhee, Mani N Nair

**Affiliations:** 1 Neurosurgery, Medstar Georgetown University Hospital; 2 Neurosurgery, Holy Cross Hospital

**Keywords:** thoracic duct injury, chyle leak, anterior spine surgery, degenerative spine disorders, octreotide, drain fluid amylase

## Abstract

Injury to the thoracic duct during anterior cervical spine surgery is a rare occurrence. A delayed chyle leak following an elective anterior cervical spinal surgery has not been reported in the literature. We present a report of a 59-year-old female with multiple prior neck surgeries who underwent an anterior cervical corpectomy and fusion (ACCF). The patient developed a delayed thoracic duct injury on postoperative day (POD) one, as no injury was noted intraoperatively. She was managed with conservative care involving a low-fat diet along with octreotide which led to the resolution of her symptoms. We present this case report because of its unique presentation and to assist spine surgeons with initial management. Surgeons should have increased awareness when performing anterior cervical approaches to the lower cervical and upper thoracic levels from the left side.

## Introduction

Thoracic duct injury is rare in spine surgery. It is often directly visualized at the time of surgery. Early recognition and management can prevent future morbidity. Reports of a direct injury to the thoracic duct are rare in the literature, and we were unable to find any case reports that detail a leak that occurred in a delayed fashion. We present the first case of a delayed chyle leak following routine anterior cervical surgery, specifically for the novelty in the delayed presentation. We propose an initial management algorithm that will assist the surgeon in routine care which will prevent an increase in patient morbidity.

## Case presentation

A 59-year-old female presented to the clinic with several months of increasing left upper extremity pain associated with numbness and tingling that was refractory to conservative management. Cardiac along with other etiologies were ruled out. She had undergone a C4-5 anterior cervical discectomy and fusion (ACDF) as well as cervical laminoplasty in the past. Her exam was significant for mild right upper extremity weakness without signs of myelopathy.

**Figure 1:**
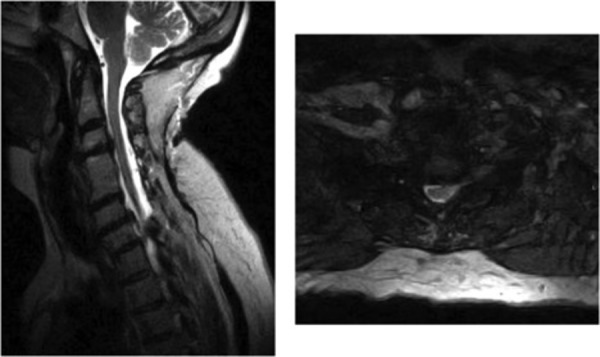
Preoperative sagittal and axial T2 MRI Preoperative sagittal and axial T2 magnetic resonance imaging (MRI) demonstrating bilateral foraminal disease at C5-6 with bilateral foraminal disease and cord impingement at C7 along with severe foraminal disease at this level on the left.

The preoperative magnetic resonance imaging (MRI) scan is shown in Figure 1. Given her prior anterior cervical surgery, an otolaryngology evaluation was performed to rule out any problems with her vocal cords. A C5-6 ACDF and C7 corpectomy were performed in an elective fashion with the insertion of an expandable cage via a left-sided approach. There were no complications during the procedure. Specifically, no milky white fluid was visualized to suggest a direct thoracic duct injury. A surgical drain was placed and the patient was closed in the standard fashion.

**Figure 2:**
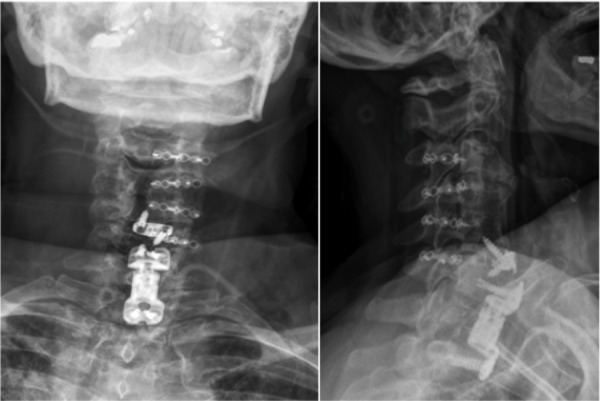
Anterior-posterior (AP) and lateral postoperative x-ray demonstrating new and old hardware with no signs of fracture or displacement

On postoperative day (POD) one, her arm pain had resolved and the numbness and strength improved. Postoperative x-rays were performed and are shown in Figure 2. After 24 hours of surgery, a milky white output into the surgical drain was apparent. The triglyceride level of the drain output was 260 mg/dl, confirming the diagnosis of a chyle leak. The patient, initially nil per os (NPO), was started on a clear liquid diet on POD three and advanced to a short-chain fatty acid diet on POD five. In addition, she initially was started on octreotide (Sandostatin, Novartis International AG, Basel, Switzerland) 100 mcg subcutaneous (SQ) ter in die (TID) at the time of diagnosis.

**Figure 3:**
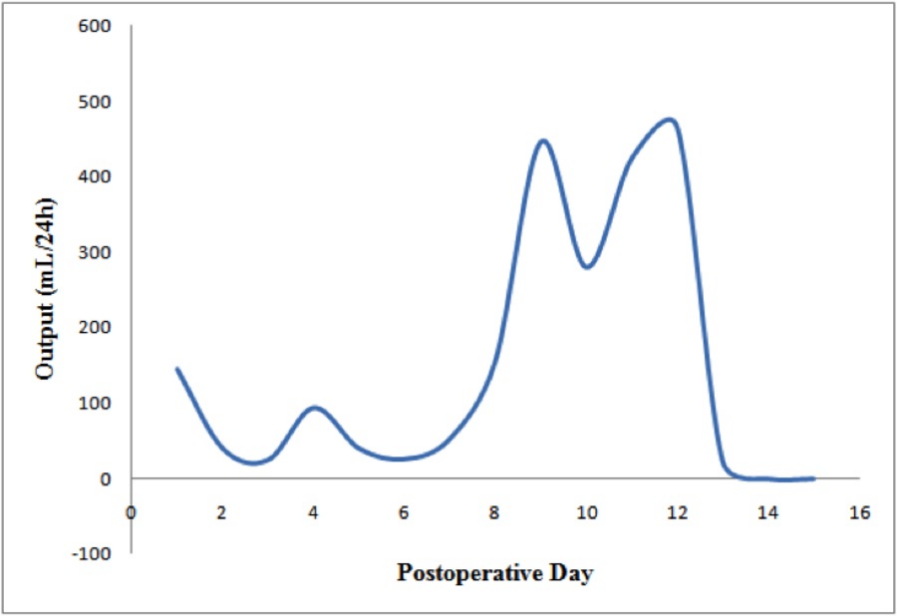
Trend in surgical drain output prior to it being removed

The trend in her drain output is shown in Figure 3. Her output eventually decreased and the remainder of her hospitalization was uneventful. She was discharged on POD 16 with plans to continue the low-fat diet for two weeks. At her two-week follow-up appointment, her pain had improved and she had no neck swelling or drainage. She continues to do well several years from surgery with no long-term complications.

## Discussion

The thoracic duct is the main conduit for lymphatic drainage [1]. The loop that it takes before entering the venous system is usually three to five centimeters above the clavicle [2-3]. The thoracic duct carries lymphatic fluid, comprised mostly of fat, from the intestinal compartment back into the bloodstream. The predominant lipid component is triglyceride ( > 200 mg/dl) in the form of chylomicrons which aids the diagnosis when chylorrhea or chylothorax is suspected [4]. Chyle has a variable milky white appearance and its production varies based on the fasting status of a patient [5-6].

Injury to the thoracic duct is a rare complication of anterior spinal surgery with few cases reported in the literature [7-8]. Most cases are seen with direct injury, meaning the injury was seen intraoperatively. This is more common with thoracic and radical neck dissections. A review of the literature did not disclose any reported cases of a thoracic duct injury that had developed in a delayed fashion, as in our patient, without obvious intraoperative injury.

**Figure 4:**
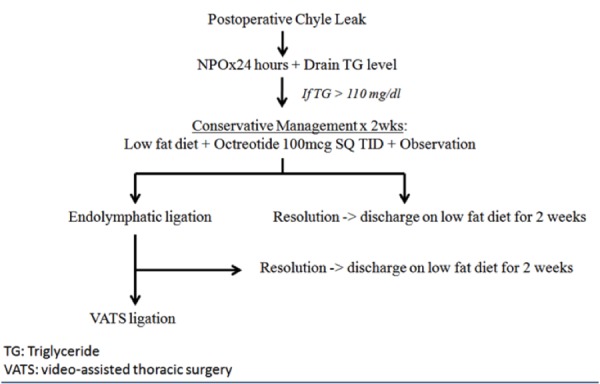
Management algorithm for use with postoperative chyle leaks

Early recognition of chyle leaks is important to prevent metabolic, nutritional, or immunological complications. Conservative management is often employed first with a 24-hour course of nothing by mouth, followed by gradual enteral formulas with low-fat content and medium to short-chain triglycerides. Octreotide is a peptide that mimics the pharmacology of somatostatin and helps to reduce chyle production and gastric secretions. Reducing the flow of lymph through the thoracic duct allows for a higher chance of spontaneous resolution. It has not been elucidated as to what rate you need to slow the flow. Anecdotal evidence has suggested that it can be beneficial in the treatment of thoracic duct injury with a low side-effect profile [9-10]. In most postoperative patients, attempting a trial of conservative management for two weeks is utilized prior to more aggressive interventions. Further research on the optimal timing of conservative management is needed. Figure 4 illustrates a simple management algorithm that can be utilized by the practicing surgeon for postoperative chyle leaks. There currently is no general consensus on the duration of conservative therapy.

## Conclusions

Thoracic duct injury during anterior cervical spine surgery is a rare occurrence and usually resolves with conservative treatment without long-term morbidity. Our case report is unique in that no direct injury was noted at the time of operation. Patients with prior anterior spinal approaches are at increased risk, necessitating added precaution by surgeons with left-sided approaches. Early recognition and use of a simple management algorithm will help to reduce potential morbidity. 
